# A comparative evaluation of the fracture resistance of three different pre-fabricated posts in endodontically treated teeth: An *in vitro* study

**DOI:** 10.4103/0972-0707.71642

**Published:** 2010

**Authors:** Prabeesh Padmanabhan

**Affiliations:** Department of Conservative Dentistry, M. R. Ambedkar Dental College, Bangalore, India

**Keywords:** AH-Plus sealer, carbon fiber post, ceramic post, panavia-F, pre-fabricated stainless steel posts

## Abstract

**Aims:**

To compare the fracture resistance and primary mode of failure of three different pre- fabricated posts like stainless steel, carbon fiber and ceramic posts in endodontically treated crowned permanent maxillary central incisors.

**Materials and Methods:**

Root canal treatment was performed on all 30 maxillary central incisors. Post space was prepared and samples were divided into three groups of 10 each. The teeth were inserted with pre-fabricated stainless steel, carbon fiber and ceramic post and cemented using adhesive resins, core fabricated and crowns placed. Mode of failure was carried out by immersing the teeth in black ink for 12 h and then sectioning them mesio-distally. Fracture above the embedded resin was considered favorable and fracture below the resin level was considered unfavorable.

**Statistical Analysis:**

Fracture strength was measured using a universal testing machine. Data were evaluated statistically using the Kruskal Wallis test and the Mann Whitney *“U”*-test. Mode of failure was evaluated statistically using the chi-square test.

**Results:**

There was a statistically significant difference showing that the stainless steel post had a better fracture resistance when compared with the other two posts and the carbon fiber showed a statistically more favorable fracture when compared with the other two posts.

**Conclusions:**

Within the limitations of this study, it can be concluded that the pre-fabricated stainless steel post exhibited a significantly higher fracture resistance at failure when compared with the carbon fiber post and the ceramic post. The mode of failure of the carbon fiber post was more favorable to the remaining tooth structure when compared with the pre-fabricated stainless steel post and the ceramic post.

## INTRODUCTION

The restoration and reinforcement of pulpless teeth is an important step in preventing fracture of the teeth. The amount of tooth structure that remains after endodontic treatment is an important consideration. Teeth critical to restorative treatment are frequently coronally deficient, yet retained by a sound root. Loss of coronal tooth structure in the badly broken down tooth requires utilization of root canal for support of crown restoration.[1] The primary objective of post and core built up is to replace the missing coronal tooth structure sufficiently to provide the required retention and resistance form for final restoration. Post and core protect or strengthen the tooth against intraoral forces by equally distributing torquing forces within the radicular dentin to supporting tissues thus dispersing forces along the root and providing retention for the core that replaced the lost coronal tooth structure and thus retain the restoration.[2]

The amount of tooth structure is a significant factor in determining the fracture resistance of an endodontically treated tooth. The strength of an endodontically treated tooth with minimal coronal tooth structure will depend heavily on post material and design. Different posts have different physical properties. An ideal post system should exhibit fracture resistance higher than the average masticatory forces.[3] In this study, the fracture resistance of the three posts was compared and evaluated to determine whether they could withstand masticatory load. There exists a definite correlation between post material and fracture of roots. Fractures above the alveolar bone were considered favorable as retreatment could be initiated due to the accessibility and the adequate amount of remaining tooth structure present to provide restorative treatment. The fracture of the post and tooth below the alveolar bone was considered unfavorable as retreatment would be difficult.[3] In this study, the primary mode of failure of the three posts were evaluated.

The aim of this study is to compare the fracture resistance and primary mode of failure of three different pre-fabricated posts, like stainless steel, carbon fiber and ceramic posts, in endodontically treated crowned permanent maxillary central incisors.

## MATERIALS AND METHODS

Thirty freshly extracted human maxillary central incisors were selected for this study. Root canal treatment was performed on all the 30 specimens. Obturation was carried out by the lateral condensation method using a 40-size gutta percha (Dentsply) as master cone.

AH-plus (Dentsply-Kronstaz, Germany), a non-eugenol endodontic sealer, was used. The crown of each tooth was reduced to a height of 1 mm above the cemento-enamel junction in order to simulate the clinical situation of a reduced tooth structure so that the resistance to fracture of the post system used would be more relevant clinically. Post space was prepared for all the 30 specimens. A post space of depth 10 mm was standardized from the cut tooth surface that was taken as the reference point.

### Grouping of teeth and post insertion

The teeth samples were divided into three groups. Each group had 10 samples.

Group 1: The post space preparation was completed with the corresponding drill provided by the manufacturer. The teeth were inserted with the pre-fabricated stainless steel post (para post, Coltene and Whaledent, USA).

Group 2: Post space preparation was completed with the corresponding drill provided by the manufacturer. The teeth were inserted with the carbon fiber post (Mirafit post, J Morita, USA).

Group 3: Post space preparation was completed with the corresponding drill provided by the manufacturer. The teeth were inserted with ceramic post (Cosmo post, Ivoclar Vivadent).

The posts were cemented using adhesive resins (Panavia F, Kuraray Co. Ltd., Japan), core fabricated and crowns placed.

### Testing procedure

All specimens were stored in saline for 24 h prior to the mechanical testing.

### Investigation of fracture resistance

A compressive load at a crosshead speed of 5 mm/min was applied using a universal loading machine at an angle of 130° to the long axis of the tooth. The load at fracture was measured and the mean was calculated using statistical analysis of Kruskal Wallis test and Mann–Whitney “*U*”–test and the significance among the three groups was analyzed.

### To investigate the mode of failure

Tooth fracture was either classified as favorable or unfavorable according to its location. Fracture above the embedded resin was considered favorable and fracture below the resin level was considered unfavorable. It was noted by two independent observers. The values were evaluated statistically using the chi-square test.

## RESULTS

In all the tested specimens fractured, Group 1 exhibited the maximum fracture resistance while Group III exhibited the least fracture resistance among the three groups [Table 1]. The Kruskal Wallis statistical test revealed that while comparing the three groups, it was found to be very highly significant at *P* <0.001.

**Table 1:** Mean and standard deviation for each group

Groups	Mean (Newtons)	Standard deviation	*P* value
Group 1 (SS)	514.1000	46.3548	0.0019
Group 2 (CF)	449.1000	28.6063	0.001
Group 3 (Ce)	362.7000	19.9947	0.0001

ss – stainless steel, cf – carbonfibre, ce -ceramic

Comparison among the individual groups was performed using the Mann–Whitney “*U*”-test.

The test showed that

- When comparing Group 1 with Group 2, it is found to be statistically highly significant.
- When comparing Group 1 with Group 3, it is found to be statistically very highly significant.
- When comparing Group 2 with Group 3, it is found to be statistically very highly significant [Table 2].

**Table 2:** Comparison among groups using Kruskal Wallis followed by Mann-Whitney ‘U’ significance difference test

Comparison of groups	Test of significance		Remarks
	Z	P	
Group 1 vs Group	2 23.099	0.0019	Highly significant
Group 1 vs Group 3	3.781	0.0001	Very highly significant
Group 2 vs Group 3	3.781	0.001	Very highly significant

Comparison of the primary mode of failure was performed among the three groups with the chi-square test and the results showed that Group 2 was more favourable than Group 1 and Group 3 [Table 3].

**Table 3:** Mode of failure among the three groups

Group	Favorable	Unfavorable
Group 1 (SS)	5	5
Group 2 (CF)	8	2
Group 3 (Ce)	2	8
Total	15	15
Chi Value X2	7.20	-
*P* value	0.0273	-

*P*<0.05

## DISCUSSION

The objective of a post-endodontic restoration is to achieve normal form and function as well as the prevention of fracture of the residual root. Other considerations are esthetics, prevention of caries and retention of final restoration.[4] The amount of tooth structure is a significant factor in determining the fracture resistance of an endodontically treated tooth. It has been shown that endodontically treated teeth restored with posts and cores of different materials and designs tend to exhibit similar fracture resistance when abundant tooth structure remains.[3]

The present study simulated a realistic condition of a reduced amount of tooth structure with a 1 mm height of coronal dentin. With such a design, the compressive load was largely borne by the post and core.[3] The purpose of this study was to compare the fracture resistance and primary mode of failure of three different pre-fabricated posts like stainless steel post (metal post), carbon fiber post (non-metal post) and ceramic post (non-metal post) in endodontically treated teeth.

Sorensen stated that tapered posts, although increasing the fracture resistance, can result in unrestorable complicated fracture upon loading. This is due to the maximum adaptation of residual root structure with a tapered post.[5] Thus, parallel-sided cylindrical posts were selected for the study.

Crown was prepared for all the samples. This was performed taking into account the fact that most of the post and core restorations are clinically followed by full-crown restorations. Cormier stated that a post and core reinforced with full-coverage crown is more fracture resistant than a post alone or a post and core combination.[6] This may be due to the fact that crowns act to distribute applied loads more evenly over the core.[7]

Mendoza,[8] in a study, showed that posts cemented with Panavia were significantly more resistant to fracture than those cemented with zinc phosphate.[8] Another study carried out by Saupe[9] has shown that microleakage associated with carbon fiber and stainless steel posts when cemented with adhesive cement (C and B Metabond and Panavia 21) were comparatively less when cemented with non-dentin bonding cements such as glass ionomer and zinc phosphate.[9] Keefe[10] performed a study to measure the bond strength and found that Panaiva 21 provided the highest bond strength for all types of post materials.[10]

The fracture resistance was evaluated using a universal load-testing machine (UTM Instron make model-1011, Frank Bacon, Michigan, USA). A compressive load at a crosshead speed of 5 mm/min was applied at an angle of 130° to the long axis of the tooth. Although, clinically, the velocity of mandibular movement varies considerably, the impact velocity of the compressive tip was maintained at a crosshead speed of 5 mm/min, which is considered as an acceptable average value.[11]

Results of the study showed that Group 1 (514.1 N) exhibited the maximum fracture resistance while Group 3 (362.7N) exhibited the least. Group 1 (pre-fabricated metallic post system) exhibited higher mean fracture resistance than Group 2 (carbon fiber post), showing that the metal post was slightly superior. Studies carried out by Purton,[12] Sidoli,[11] Love[13] and Gallo[14] comparing these two post systems have shown similar results. Group 2 (carbon fiber post) when compared with Group 3 (ceramic post) showed that the carbon fiber post exhibited higher fracture resistance than the ceramic post. Different studies performed by Maccar,[5] Cormier,[6] Fokkinga[15] and Mannocci. F[16] comparing these two post systems have shown similar results. Group 1 (pre-fabricated metallic post system) when compared with Group 3 (ceramic post) showed that the metal post exhibited a higher fracture resistance than the ceramic post. Various studies performed by Purton,[17] Cohen[18] and Dietschi[19] comparing these two post systems have shown similar results.

The primary mode of failure was evaluated at the above-mentioned fractured loads. The level of embedding of the tooth sample in the autopolymerizing resin was 3 mm below the cemento-enamel junction, which simulated the level of alveolar bone. The mode of failure was considered favorable or unfavorable depending on whether the fracture of the tooth sample was above or below the embedded resin, respectively. Fractures above the embedded resin were considered favorable as retreatment could be initiated due to the accessibility and the adequate amount of remaining tooth structure present to provide restorative treatment. The fracture of the sample below the embedded resin was considered unfavorable as retreatment would be difficult.[3] The results showed that of 10 samples in each group, unfavorable fractures in the pre-fabricated parapost (Group 1) were five, carbon fiber (Group 2) was two and ceramic (Group 3) was eight. The results suggested that when the carbon fiber post systems were used, less damage to the tooth structure occurred at failure load. The results obtained in this study resembled the study carried out by Dean,[20] which compared stainless steel post and carbon fiber post. The results showed that of 10 samples, unfavorable fractures in stainless steel were five and carbon fiber was one.[20]

Another study was carried out by Ferrari,[16] which compared carbon fiber and ceramic post. The results showed that of 10 samples, unfavorable fracture in carbon fiber was one and ceramic was six.[16] The results of the present study show that even though the fracture resistance of the carbon fiber post was inferior to the stainless steel post, they performed superiorly when facture mode was taken as a parameter.

There exists a definite correlation between post material and fracture of roots. The post material should have the same modulus of elasticity as the root dentin to distribute the applied forces evenly along the length of the post and the root. Studies have shown that when a system with components of different rigidity is loaded, the more-rigid component is capable of resisting forces without distortion. The less-rigid component fails and relieves stresses.[21] Post with modulus of elasticity significantly greater than that of dentin might create stresses at the tooth/cement/post interface, with the possibility of post separation and failure. Modulus of elasticity of dentin is approximately 14–18 GPa. According to the manufacturers of the material included in this study, the carbon fiber posts have modulus that are approximately 9–50 GPa. This similarity in elasticity may allow post flexion to mimic tooth flexion. The carbon fiber post absorbs and distributes the stresses and thus shows reduced stress transmission to the root.[6] The longitudinal arrangement of fibers in the carbon fiber post and the modulus of elasticity of the post that is less than or equal to that of the dentin may redistribute the stress into the tooth and away from the chamfered shoulder to increase the likelihood of failure of the post core\root interface instead of root fractures.[22] In contrast, the parapost has got a modulus of elasticity that is 8–9 times that of dentin.[6] The higher Youngs modulus (rigidity) of the metallic post system makes it stiff and unable to absorb stresses. In addition, transmission of occlusal and lateral forces through a metallic core and post can concentrate stresses, resulting in the possibility of unfavorable fracture of the root.[23]

Modulus of elasticity of ceramic is 170–213 GPa, which is approximately 15-times that of dentin.[6] Ceramic posts are too rigid and transmit more stress to the root canal than the fiber posts, which lead to irreversible root damage.[24]

An ideal post system should exhibit fracture resistance more than the average physiologic masticatory forces, but should not be too high to cause catastrophic fracture of the root. Fracture resistance is of greater importance than retention because post can be recemented if dislodged from the tooth. However, if the root fractures, the tooth is invariably lost.[24]

The other factors that affect the fracture resistance of endodontically treated teeth are post diameter, length, design and adaptability, amount of remaining dentin, cement and method of cementation, core material and design, crown design and biocompatibility of post material.[1]

The result obtained from this study should be interpreted with caution, as teeth were mounted for load testing in materials that have limited resiliency. This takes out the viable periodontal ligament and resilient alveolar bone out of equation, which are crucial parameters on loading.[1] Fracture resistance was studied on universal testing machines, which did not take into account the oblique, torsional and lateral shearing forces produced during chewing. Another factor is the multidirectional characteristics of masticatory forces, which cannot be duplicated in such machines where a single unidirectional load is applied.[1] It is clear that this type of *in vitro* loading does not represent the complete situation *in vivo*. However, it was focused on the basis of tests previously reported in the literature involving post and core systems.

## CONCLUSIONS

According to the findings and within the limitations of this study, it can be concluded that

- The study showed that with a single-angle compressive load, teeth restored with the pre-fabricated stainless steel post (parapost) exhibited significantly higher fracture resistance at failure when compared with the carbon fiber post and the ceramic post.
- The mode of failure of the carbon fiber post with angled compressive load testing, however, was more favorable to the remaining tooth structure when compared with the pre-fabricated stainless steel post and the ceramic post.
